# Unexpected increase in the bone marrow toxicity of mitomycin C (MMC) – reply

**DOI:** 10.1054/bjoc.2000.1670

**Published:** 2001-03

**Authors:** Y Imai, T Hagiwara


doi: 10.1054/ bjoc.2000.1670, available online at http://www.idealibrary.com on IDEAL

### Unexpected increase in the bone marrow toxicity of mitomycin C (MMC) – reply

The supplier of Mitomycin C (Kyowa Hakko) was invited to respond to the above Letter to the Editor.

In their Letter to the Editor, Yoshimoto and colleagues conclude that 'MMC should be used with caution whatever the reason for its increase in toxicity'. We feel apprehensive, however, that 'the increase in toxicity' as argued by the authors might be subjective rather than objective for the following reasons:

1. Quality control of MMC.

We are confident that the manufacture of MMC has been consistently carried out under stringent quality control. Our MMC has passed the inspection of the MCA (UK) and the FDA (USA) as raw material and of the MCA as a pharmaceutical product. The authors cited test results of the quality, in vitro activity and in vivo marrow depression of MMC. The tests were conducted by our company due to the repeated requests of Dr Yoshimoto and his colleagues, who suspected the change of MMC itself as the cause of the claimed increase in toxicity. We have disclosed our test results to them, which showed, as they cite, no difference between production lots of MMC manufactured in different years. Moreover, our database covering all adverse reaction reports within Japan during the controversial period reveals no increase of severe marrow depression with MMC. Therefore, Kyowa Hakko does not accept any association between the quality of MMC and an increase or enhancement of marrow depression.

2. Is the claimed increase related to data analysis?

The authors list patient age, disease stage and dose or dosing schedule as possible covariates. The patients with previous treatment with anticancer drugs and those with a past history of diseases that might affect bone marrow function were excluded according to the figure legend. However, other covariates such as sampling frequency and interval could be important in interpreting the data. Yoshimoto et al have not presented the sampling frequency in a particular period after administration (such as until the next CMF therapy) or sampling interval in the period during which blood cells are expected to reach true nadir.

We invite Yoshimoto and his co-authors to provide their raw data for analysis by a medical statistician.

Overall, we do not consider that there is genuine evidence that MMC has caused an increase of adverse reactions.

*Y Imai and T Hagiwara*

*© 2001 Cancer Research Campaign*

